# Supplementary material for: Effects of Trichoderma harzianum combined with Phanerochaete chrysosporium on lignin degradation and humification during chicken manure and rice husk composting
Source: Front Microbiol. 2025 Feb 28;16:1515931. doi: 10.3389/fmicb.2025.1515931 (PMC11906335; doi:10.3389/fmicb.2025.1515931)
Supplement: Supplementary file 1 [file Data_Sheet_1.zip › 6.Function/All/FunGuild/l1/PLSDA-score.FunGuild_l1.All.pdf]

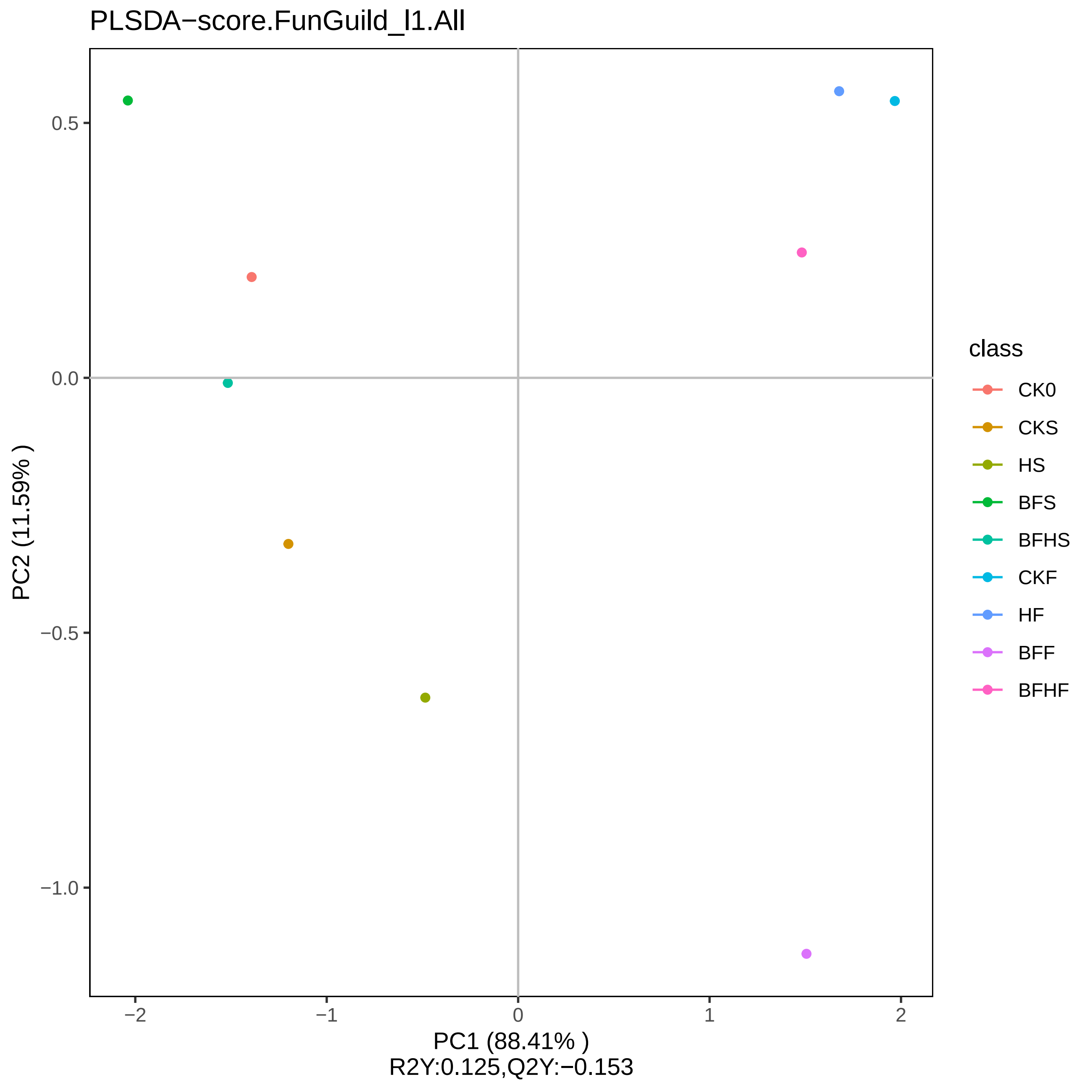
PLSDA−score.FunGuild_l1.All
0.5
0.0
−0.5
−1.0
PC2 (11.59%)
−2
−1
0
1
2
PC1 (88.41%)
R2Y:0.125,Q2Y:−0.153
class
CK0
CKS
HS
BFS
BFHS
CKF
HF
BFF
BFHF
